# Uncovering the Dynamics of Cardiac Systems Using Stochastic Pacing and Frequency Domain Analyses

**DOI:** 10.1371/journal.pcbi.1002399

**Published:** 2012-03-01

**Authors:** Mathieu Lemay, Enno de Lange, Jan P. Kucera

**Affiliations:** 1 Department of Physiology, University of Bern, Bern, Switzerland; 2 Cardiovascular Research Laboratory, David Geffen School of Medicine, University of California, Los Angeles, Los Angeles, California, United States of America; University of California San Diego, United States of America

## Abstract

Alternans of cardiac action potential duration (APD) is a well-known arrhythmogenic mechanism which results from dynamical instabilities. The propensity to alternans is classically investigated by examining APD restitution and by deriving APD restitution slopes as predictive markers. However, experiments have shown that such markers are not always accurate for the prediction of alternans. Using a mathematical ventricular cell model known to exhibit unstable dynamics of both membrane potential and Ca^2+^ cycling, we demonstrate that an accurate marker can be obtained by pacing at cycle lengths (CLs) varying randomly around a basic CL (BCL) and by evaluating the transfer function between the time series of CLs and APDs using an autoregressive-moving-average (ARMA) model. The first pole of this transfer function corresponds to the eigenvalue (λ_alt_) of the dominant eigenmode of the cardiac system, which predicts that alternans occurs when λ_alt_≤−1. For different BCLs, control values of λ_alt_ were obtained using eigenmode analysis and compared to the first pole of the transfer function estimated using ARMA model fitting in simulations of random pacing protocols. In all versions of the cell model, this pole provided an accurate estimation of λ_alt_. Furthermore, during slow ramp decreases of BCL or simulated drug application, this approach predicted the onset of alternans by extrapolating the time course of the estimated λ_alt_. In conclusion, stochastic pacing and ARMA model identification represents a novel approach to predict alternans without making any assumptions about its ionic mechanisms. It should therefore be applicable experimentally for any type of myocardial cell.

## Author Summary

Cardiac arrhythmias are frequent complications of heart disease and an important cause of morbidity and mortality. The rhythmic activity of the heart relies on the action potential, a bioelectrical signal characterized by complex dynamics involving ion currents and intracellular calcium cycling. When these dynamics become unstable, arrhythmogenic patterns can emerge. A typical example is the beat-to-beat alternation of action potential parameters, a phenomenon called alternans, which represents a well known mechanism precipitating severe arrhythmias. Alternans results from the interaction of action potentials during consecutive beats. Classically, this interaction is investigated by describing the dependence of action potential parameters on previous diastolic intervals and action potential durations. However, experiments have shown that quantitative markers derived in this way are only approximate or even inappropriate to predict alternans. Here, we devised a novel procedure for the reliable prediction of alternans, based on introducing small random variations of pacing intervals followed by signal processing in the frequency domain. Using a biophysical model of the cardiac cell, we demonstrate that our algorithm accurately predicts the onset of alternans during pacing at an accelerating rate or during the application of a drug. Our approach may thus open new perspectives for the clinical evaluation of arrhythmias.

## Introduction

In cardiac physiology, alternans designates the alternation of action potential (AP) parameters (e.g., AP duration (APD), calcium transient) from beat to beat [1], [2]. It leads to dispersion of refractoriness and represents a well established mechanism of conduction block and thus of severe reentrant arrhythmias [3], [4]. At the cellular level, alternans results from complex dynamic interactions between membrane potential (V_m_), ion currents and intracellular calcium cycling, which can together lead to different types of dynamical instabilities [2], [5]–[8].

The classical understanding of the genesis of alternans is based on the concepts of restitution functions and iterated map models [5], [9]–[11]. In the classical theory [5], [9], the relation between APD and the previous DI is first characterized by the APD restitution function *f* as APD = *f*(DI). During pacing at a given basic cycle length (BCL), the next DI can then be inferred as BCL–APD and the next APD mapped using *f*. By iteration, successive APDs and DIs can be reconstructed. The steady state is determined by the intersection of the line APD = BCL – DI with *f*. If the slope of the restitution function α = d*f*/dDI is <1 at this point, the system is stable, and if α>1, the system is unstable. Because of the nonlinear nature of *f*, this instability results in alternans, period doubling cascades and chaos via a variety of dynamical routes [12]–[14].

However, it has been shown that the criterion α = 1 for the onset of alternans is only approximate or even inappropriate. Indeed, alternans can be present even if α<1, or conversely, APD may not alternate although α>1 [15]–[18]. These discrepancies can be explained by the notion of “memory” [5], [16], [19]–[23], reflecting the fact that APD depends not only on the previous DI, but on several previous DIs and APDs. In multicellular tissue, these discrepancies can also be explained by the fact that electrotonic interactions and a steep conduction velocity restitution relation can further affect the APD restitution slope at which alternans occurs by exerting important stabilizing or destabilizing effects [15], [16], [24].

In experiments, restitution is conventionally investigated by pacing at steady-state and by introducing premature or delayed stimuli (S1S2 protocol, S1S2 restitution curves), or by decreasing BCL stepwise and examining steady-state APD vs. DI at the end of each step (dynamic restitution curves). Results at odds with the classical theory have then motivated researchers to develop refined pacing protocols and analyses incorporating the notion of memory to investigate alternans, such as the “perturbed downsweep protocol” [17], [20], [21], [25]. To untangle the effects of memory, recent studies used pacing protocols in which the DI is varied slowly in a sinusoidal manner, revealing hysteresis between APD and DI [26], or in a random manner, minimizing the influence of previous APDs and DIs on the current pacing cycle [22]. With this latter protocol, it was also demonstrated using regression analysis that APD depends on the APDs and DIs during several previous cycles [27].

In mathematical cardiac cell models, seminal insights have been obtained using eigenmode analysis [28], [29], in which one considers the deviation of the time course of model parameters from their steady-state periodic time course during pacing at a given BCL. This deviation is then decomposed as a sum of eigenmodes associated to corresponding eigenvalues (λ). For every eigenmode, the time course of model variables is scaled by the corresponding λ after each pacing cycle. When |λ|<1, the eigenmode is attenuated from beat to beat and eventually dissipates. In contrast, the eigenmode is amplified when |λ|>1, which implies instability. When λ is a real number, the sign of λ associates the corresponding eigenmode with either memory (λ>0, same polarity for every beat) or alternans (λ<0, polarity changes every beat). Thus, at least in single cell models, eigenmode analysis formally defines the *exact criterion* for the onset of alternans as λ_alt_ = min{Re(λ)} = −1. However, eigenmode analysis requires accessing the internal model variables and, therefore, it is not feasible experimentally. Thus, applications of eigenmode analysis have so far been limited to computer simulation studies [30], [31] and no straightforward approach has been designed on this basis to predict alternans in an experimental setting.

In previous work [24], we introduced the concept of cardiac tissue as a “filter” transforming an input (e.g., a series of pacing intervals varying stochastically) into an output (e.g., the series of APDs or DIs). We examined the filter characteristics in the frequency domain in terms of gain and phase shift using the transfer functions between the series of pacing intervals and the series of APDs (H_t→a_) and between the series of pacing intervals and the series of DIs (H_t→d_), respectively. In the present study, we developed a generalized framework for a straightforward and accurate prediction of alternans. We devised an approach permitting to quantify the eigenvalue λ_alt_ and these transfer functions by using only experimentally measurable quantities (APD, DI) without the requirement to access internal model variables. The first step of this approach consists of using pacing intervals varying stochastically around a mean BCL. In the next step, the poles (including λ_alt_) and zeros of the transfer functions H_t→a_ and H_t→d_ are identified by fitting an autoregressive-moving-average (ARMA) model to the recorded values of APD and DI [32].

The power of this approach was evaluated in the cardiac cell model of Sato et al. [8]. This model is formulated in three versions (based on different sets of model parameters) implementing the following mechanisms of alternans: 1) V_m_-driven (alternans attributable to the gating kinetics of membrane ion channels, with a steep APD restitution curve), 2) Ca^2+^-driven with positive Ca^2+^ to APD coupling (large Ca^2+^ transients generating longer APDs), and 3) Ca^2+^-driven with negative Ca^2+^ to APD coupling (large Ca^2+^ transients generating shorter APDs). In the latter two versions, alternans originates from an instability of Ca^2+^ cycling and occurs even in the presence of shallow APD restitution curves, thus reproducing recent experimental and theoretical findings [6], [7], [33]. The three versions of the Sato et al. model thus offered the advantage to test our approach for three fundamentally different ionic mechanisms of alternans. For this purpose, we compared the marker λ_alt_ obtained using ARMA model identification during stochastic pacing with the exact control value of λ_alt_ derived using eigenmode analysis. Our results provide the proof of principle that the eigenvalue λ_alt_ can be estimated from the time series of pacing cycle lengths, APDs and DIs, and thus that the criterion λ_alt_ = −1 could be utilizable experimentally.

## Methods

### Cell model and computer simulations

We used the model of Sato et al. [8], who combined the Fox et al. canine ventricular myocyte model [34] with the model of intracellular cycling proposed by Shiferaw et al. [33]. As mentioned above, this model is formulated in three versions implementing different mechanisms of alternans, including alternans originating from an instability of Ca^2+^ cycling.

The three different versions of the model were stimulated with 1-ms current pulses of 50 µA/µF as in the original study of Sato et al. [8], which corresponded approx. to 1.23 times diastolic threshold. Simulations were run with a constant time step of 0.005 ms. Gating variables were integrated using the method of Rush and Larsen and other model variables were integrated using the forward Euler method. Activation time was defined at depolarization to −35 mV and repolarization time at −85 mV, respectively. APD was defined as the interval between activation and repolarization times and corresponded approximately to APD at 92% of repolarization. Intervals between successive activations were equivalent to pacing intervals due to the short and quasi constant latency in response to stimulation.

### Eigenmode analysis (using internal model variables), derivation of transfer functions, and link with classical restitution slopes, alternans and memory

In all ionic cardiac cell models, including the Sato et al. model, the state of the cell at any time *t* is fully described by a vector **v** of *N* linearly independent variables (*N* = 16 in the Sato et al. model), and the temporal evolution of the model is described by d**v**/d*t*, which is defined as a function of **v**. *N* defines the order of the cell model.

At a given basic cycle length (BCL), there exists a unique function that maps **v** *_i_* (**v** at the onset of the *i* ^th^ stimulus) to **v** *_i_*_+1_ (at the onset of the *i*+1^th^ stimulus). At steady state, **v** maps onto itself, defining the steady state vector **v**_BCL_. As shown by Li and Otani [28], the mapping function can be linearized near steady state as
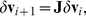
(1)where δ**v** = **v**−**v**_BCL_ is a small perturbation of **v**_BCL_ and **J** is the Jacobian matrix of this mapping. The element of **J** in column *c* and row *r* is defined as
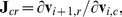
(2)where **v** *_i_*_+1,*r*_ is the *r* ^th^ element of **v** *_i_*_+1_ and **v** *_i_*_,*c*_ is the *c* ^th^ element of **v** *_i_*. In our simulations, **J** was computed by introducing a small perturbation δ of the corresponding element of **v**_BCL_, applying the modified ***v*** as initial condition and by evaluating ∂**v** near the limit δ→0 after running the model for one BCL.

As shown previously [28], the response at BCL is stable if the eigenvalues λ of **J** all lie within the unit circle in the complex plane (<1 in absolute value), and the onset of alternans coincides exactly with one of the eigenvalues (λ_alt_) being equal to −1.

#### Derivation of transfer functions

To derive the transfer function between a time series of pacing cycles fluctuating around BCL and APD, we first need to define and compute a vector **a** that describes the deviation of the *i* ^th^ APD (*a_i_*) from its steady state value (*a*_BCL_) in response to a perturbation δ**v** *_i_*, according to the inner product
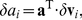
(3)where the superscript ^T^ denotes transposition. Similar to the computation of **J**, individual elements of **a** were computed in the simulations by introducing a small perturbation δ of the corresponding element of **v**_BCL_, by applying the modified **v** as initial condition to simulate the AP, and by evaluating APD near the limit δ→0. We also linearize the change of **v** resulting from a small deviation δ*t_i_* of the *i* ^th^ pacing interval (preceding the *i*+1^th^ AP) from BCL as
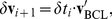
(4)where **v**′_BCL_ = d**v**/d*t* immediately prior to the onset of stimulation during steady state pacing at BCL. By combining Eqs. 1 and 4, we can now describe the behavior of the model in the presence of deviations δ*t*_i_ of the pacing cycle length from BCL as
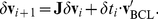
(5)Application of the *Z*-transform on Eq. 5 gives:
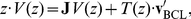
(6)where *V*(*z*) and *T*(*z*) are the *Z*-transforms of δ**v** and δ*t*, respectively. Solving for *V*(*z*) gives
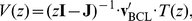
(7)where **I** is the identity matrix, and by virtue of the *Z*-transform of Eq. 3, *A*(*z*) = **a** ^T^ *V*(*z*), we obtain
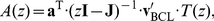
(8)where *A*(*z*) is the *Z*-transform of δ*a*.

The transfer function H_t→a_(*z*) between pacing cycles and APD is therefore
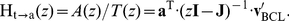
(9)The inversion of *z* **I**–**J** is rendered more tractable by diagonalization of **J** as **J** = **EDE** ^−1^, where **E** is formed by the eigenvectors of **J** (in the columns of **E**) and **D** is a diagonal matrix formed by the corresponding eigenvalues λ:
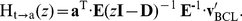
(10)In Eq. 10, (*z* **I**–**D**)^−1^ is a diagonal matrix with elements of the form 1/(*z*−λ). By explicitly developing the multiplications in Eq. 10, H_t→a_(*z*) can finally be expressed as a fraction of two polynomials:
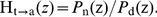
(11)The zeros of H_t→a_(*z*) are the roots of the numerator *P*_n_(*z*), which is maximally of the *N* ^th^ degree, and the poles of H_t→a_(*z*) are the roots of the denominator *P*_d_(*z*) and correspond to the *N* eigenvalues of **J**. In our simulations, these eigenvalues and the roots of polynomials were computed using established numerical procedures [35].

The transfer function H_t→d_(*z*) between pacing cycles and diastolic intervals can easily be derived from the fact that δ*t_i_* = δ*a_i_*+δ*d_i_*. Therefore, *T*(*z*) = A(*z*)+*D*(*z*) (*D*(*z*) being the *Z*-transform of δ*d*) and
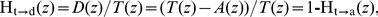
(12)which can also be expressed as a ratio of polynomials permitting the computation of its zeros (the poles remain equal to the eigenvalues).

The frequency response in terms of gain and phase shift, corresponding to the ratio of the discrete-time Fourier transforms of the output (APDs or DIs) and the input (cycle lengths) is obtained by substituting *z* = 2π*if* in the above equations, with *f *∈ [0, 0.5]. *f* is expressed in *beat^−1^*, with *f* = 0.5 beat^−1^ corresponding to the frequency of alternans (once every 2 beats).

#### Link with classical restitution slopes

The S1S2 restitution slope S_S1S2_ simply corresponds to
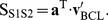
(13)The dynamic restitution slope S_dyn_ can be inferred from the steady state response to a constant and continuous change δ*t* of BCL, i.e., to a sustained change at a frequency *f* = 0. In the *Z*-domain, *f* = 0 corresponds to *z* = *e* ^2π*if*^ = 1, and S_dyn_ corresponds to
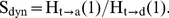
(14)


### Autoregressive-moving-average (ARMA) modeling analysis (based on the memory of previous pacing cycles), derivation of transfer functions, and link with classical restitution slopes and alternans

#### Derivation of transfer functions using the memory model of previous pacing cycles

The derivation presented above, based on eigenmode analysis, requires the knowledge of the internal variables of the cellular model. However, in a practical setting, only a restricted set of quantities are measurable, such as APD and DI. As proposed previously, the notion of memory in cardiac tissue can be approached by considering that APD depends not only on the previous DI but on several previous APDs and DIs [21], [25]:
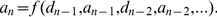
(15)Because pacing cycle lengths *t_n_* are linked to *a_n_* and *d_n_* by *t_n_* = *a_n_*+*d_n_*, Eq. 15 can also be formulated as
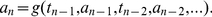
(16)Linearizing Eq. 16 around steady state at BCL for small variations δ*a_n_* and δ*t_n_*, we obtain
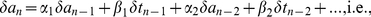
(17)

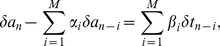
(18)where *M* is the number of previous cycles accounted by memory. Eq. 18 represents an autoregressive-moving-average model (ARMA) with δ*t_n_* as input series and δ*a_n_* as output series. Its transfer function is easily obtained using Z-transformation [32]:
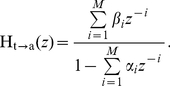
(19)From Eq. 19, we see that H_t→a_(*z*) is also expressed as a ratio of polynomials *P*_n_(*z*)/*P*_d_(*z*) and that its poles and zeros can be obtained by finding the roots of *P*_n_(*z*) and *P*_d_(*z*).

Comparing the result of the previous section (Eq. 11) with Eq. 19, we first conclude that the transfer functions H_t→a_(*z*) and H_t→d_(*z*) or the corresponding sets of poles and zeros *implicitly* contain all the information about both alternans and memory in the framework of restitution. Second, we conclude that memory (in the sense of Eq. 15) is an implicit characteristic of any model of order *N*>1, and that in theory, the number of memory cycles *M* is at most equal to the order of the model *N* (*M*≤*N*).

It must however be noted that modern cardiac cell models always exhibit features with very short time scales (e.g., the gates of the Na^+^ current) as well as features with considerably longer time scales (e.g., the filling of the sarcoplasmic reticulum with Ca^2+^). Thus, mathematically speaking, cellular models of cardiac electrical activity belong to the category of stiff systems, for which it is known that some components dissipate considerably faster than others. These rapidly dissipating components correspond to eigenmodes which have eigenvalues close to 0. It then appears likely that all these models will have only few significant eigenmodes. Indeed, in the Beeler-Reuter ventricular cell model [36], Li and Otani observed that a majority of eigenvalues lie close to 0 and they found only 2 significant eigenmodes [28]. Therefore, in practice, a majority of poles and zeros will lie close to 0 and will exert only insignificant influences on the transfer functions. Consequently, low values of *M*<<*N* with only a few coefficients α and β may suffice to describe the dynamic behavior of a given model.

#### Link with classical restitution slopes

The S1S2 restitution slope S_S1S2_ corresponds to
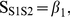
(20)and from Eqs. 12, 14, and 19, S_dyn_ is related to the coefficients α*_i_* and β*_i_* of the polynomials *P*_n_(*z*) and *P*_d_(*z*) as
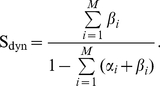
(21)In this framework, the “memoryless” map APD = *f*(DI) [9] with α = *d*APD_BCL_/*d*DI_BCL_ represents a particular case with α_1_ = −α, β_1_ = α, and α_2_ = α_3_ = … = β_2_ = β_3_ = … = 0, for which the alternans criteria S_dyn_ = 1, S_S1S2_ = 1 and λ_alt_ = −1 are all equivalent.

### Test protocols with the cell model, ARMA model identification and data analysis

For each BCL tested (in decremental steps of 5 ms from 1000 ms to 500 ms, and then in steps of 1 ms), the 3 versions of the Sato et al. model were paced at this BCL until a steady state 1∶1 response was obtained or until sustained alternans was documented. Because our goal was to be as close as possible (within a reasonable computational limit) to the true steady state when considering all model variables, steady state was considered to be attained when the relative beat to beat variation of all model variables was <10^−7^. Steady state defined according to this criterion was obtained after 150–1000 beats. In presence of a stable 1∶1 response, the following protocols and analyses were conducted:

1. **Eigenmode analysis:** The transfer functions H_t→a_ and H_t→d_ were computed as described above (Eqs. 1–12). The eigenvalue closest to −1 was defined as λ_alt_, and S_S1S2_ and S_dyn_ were derived according to Eqs. 13 and 14. The eigenvalue closest to +1 was defined as the principal memory eigenvalue, λ_mem_.
2. **Conventional pacing protocols:** Steady state DIs and APDs were used to construct the dynamic restitution curve and to measure S_dyn_. Classical S1S2 stimulation protocols were used to construct S1S2 restitution curves for various BCLs (i.e., S1S1 intervals) and to measure S_S1S2_. For every BCL at which the S1S2 protocol was conducted, steady state conditions were first obtained as described above.
3. **Stochastic pacing protocol and ARMA model identification:** After having reached steady state, the cell model was paced at cycle lengths (CLs) varying randomly around BCL with a Gaussian distribution having a predefined standard deviation (SD). The best coefficients α and β relating APD and cycle lengths according to an ARMA model (Eq. 18) of predefined order *M* (2 or 3) were identified using mean square error minimization [32] in series of 30–100 cycles. The computed coefficients were used to estimate the transfer functions H_t→a_ and H_t→d_ together with their poles and zeros. S_S1S2_ and S_dyn_ were estimated according to Eqs. 20 and 21. H_t→a_ and H_t→d_ were also computed directly from the discrete Fourier transforms (DFT) of APDs, DIs and pacing cycle lengths (CL) as H_t→a_(*f*) = DFT(APD)/DFT(CL) and H_t→d_(*f*) = DFT(DI)/DFT(CL).

The results of these three analyses were compared and evaluated in terms of the ability of S_S1S2_, S_dyn_ and λ_alt_ to predict the onset of alternans in the different versions of the Sato et al. model. In some simulations, a random error was added to APD to mimic experimental measurement error.

## Results

### Occurrence of alternans, main eigenvalues and rate adaptation in the three model versions

**Figure 1:**
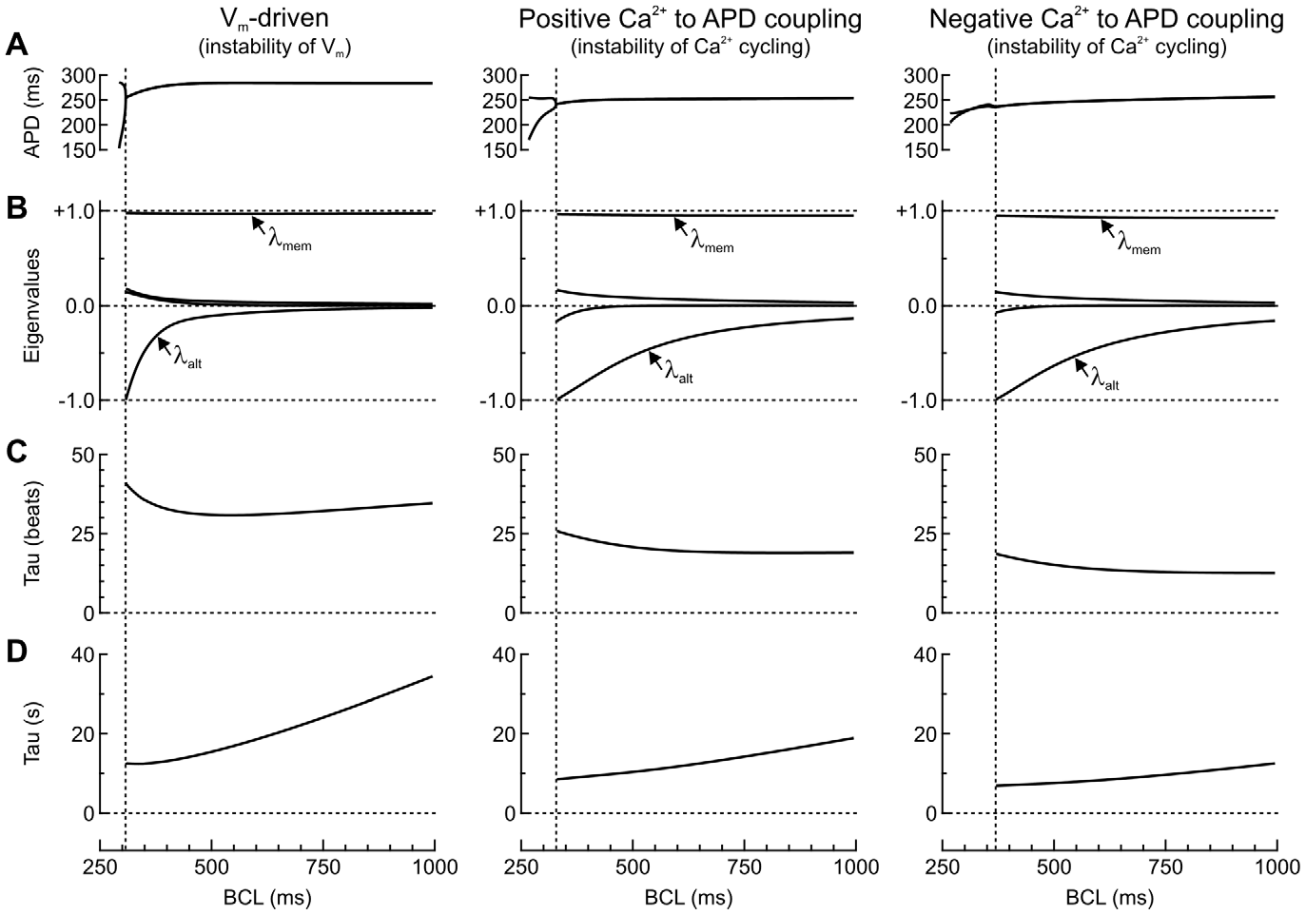
Bifurcation to alternans and eigenvalues in the three model versions. *A*: APD vs. BCL at steady state pacing (relative beat to beat variation of model variables <10^−7^). The vertical dotted lines denote the bifurcations to alternans. *B*: Eigenvalues with an absolute value >0.1 vs. BCL. The eigenvalue closest to −1 is the alternans eigenvalue λ_alt_. The eigenvalue closest to +1 is the principal memory eigenvalue λ_mem_. *C*: Time constant of memory (expressed in number of beats, Eq. 23). *D*: The same time constant expressed in units of time (Eq. 24).

Figure 1 *A* depicts steady-state APD vs. BCL in the three versions of the Sato et al. model and illustrates the bifurcations to alternans. The shortest BCLs at which alternans was absent during steady state pacing were 308 ms, 330 ms and 370 ms in the V_m_-driven model, the Ca^2+^-driven model with positive Ca^2+^ to APD coupling and the Ca^2+^-driven model with negative Ca^2+^ to APD coupling, respectively. Shortening these BCLs by 1 ms resulted in sustained alternans.

In Figure 1 *B*, the corresponding eigenvalues with an absolute value >0.1 are represented. As predicted by eigenmode analysis [28], the onset of alternans occurred exactly when λ_alt_. reached −1 in all three model versions. The principal memory eigenvalue λ_mem_ remained close but always less than +1 at all BCLs tested. All the other eigenvalues were close to 0. Eigenvalues with an absolute value <0.1 (not shown) correspond to eigenmodes which dissipate by >99% after 2 beats, and which therefore have only very small influences on the dynamics of the model.

The time course with which the model stabilizes towards steady state can be inferred from λ_mem_. Because λ_mem_ is the eigenvalue closest to 1 (in absolute value), it determines the slowest time scale in the model. During pacing at a given BCL, the corresponding eigenmode (E_mem_) decays as
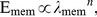
(22)where *n* is the number of beats. The time constant of this process is
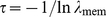
(23)(expressed in number of beats), i.e.,
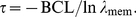
(24)in absolute time units. This time constant is shown in Figure 1 *C* and *D*. Thus, in the Sato et al. model, accommodation of model variables (and thus of APD) to a given pacing rate occurs with a time constant in the range of 10–40 beats, which corresponds to 8–40 s, depending on BCL.

### Restitution curves in the three model versions

**Figure 2:**
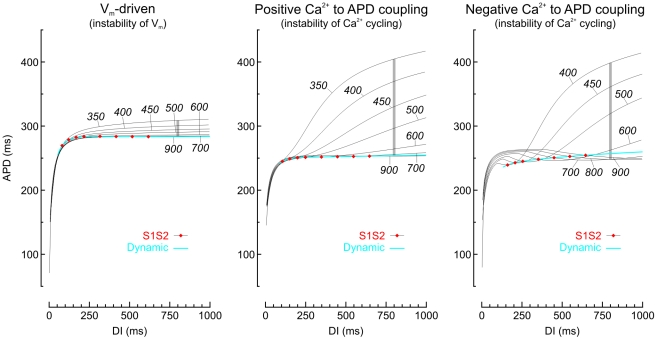
Dynamic and S1S2 restitution curves in the three model versions. Each panel represents the dynamic restitution curve, i.e., steady-state APD vs. DI (blue) and several S1S2 restitution curves (labels indicate BCL, i.e., the S1S1 pacing cycle length). For each S1S2 restitution curve, a broad range of S1S2 intervals were explored, yielding DIs from 0 to 1000 ms. The intersections of the S1S2 restitution curves with the dynamic restitution curve are marked with red diamonds. The vertical gray bars denote memory amplitude at DI = 800 ms.

Figure 2 illustrates dynamic and S1S2 restitution curves obtained using conventional pacing protocols. The dynamic restitution curves were generated from the steady state APD and DI values at each individual BCL (i.e., at each S1S1 pacing cycle length). Each S1S2 restitution curve was then obtained by introducing a modified cycle length (S1S2 interval) and by representing the APD of the subsequent AP vs the previous DI. With this approach, a family of S1S2 restitution curves was obtained.

In all versions of the model, S1S2 and dynamic restitution functions never overlapped. As shown previously [19], this absence of overlapping proves that APD depends on more parameters than the previous DI. In the V_m_-driven model, the S1S2 restitution curves formed the closest pattern gathered around the dynamic restitution curve, and the S1S2 restitution curves were always monotonically increasing. In contrast, in the two Ca^2+^-driven versions of the model, the S1S2 restitution curves deviated substantially from the dynamic restitution curve, especially at larger DIs. In the Ca^2+^-driven model with positive Ca^2+^ to APD coupling, the prominent increase of APD at long DIs was the consequence of larger Ca^2+^ transients, resulting in AP prolongation. In the Ca^2+^-driven model with negative Ca^2+^ to APD coupling, the S1S2 curves were non monotonic and each curve exhibited a segment with a negative slope. This phase of decreasing APD with increasing DI was the consequence of larger Ca^2+^ transients, resulting in this case in AP shortening.

Cherry and Fenton [23] introduced the concept of “memory amplitude” as a measure of short term memory. This measure is defined as the range of APD values covered by the S1S2 restitution curves at a predefined long DI. For DI = 800 ms, memory amplitude was 25, 152 and 151 ms in the V_m_-driven and the Ca^2+^-driven model versions with positive and negative Ca^2+^ to APD coupling, respectively. According to this criterion, the Ca^2+^-driven models exhibit a larger amount of short term memory compared to the V_m_-driven model. However, memory amplitude and λ_mem_ cannot be compared directly, because the former reflects APD changes over 2 (or a very few) beats, whereas the latter reflects the longest time scale of the model dynamics.

### Example analysis using ARMA model identification and transfer functions in the Ca^2+^-driven model with positive Ca^2+^ to APD coupling

**Figure 3:**
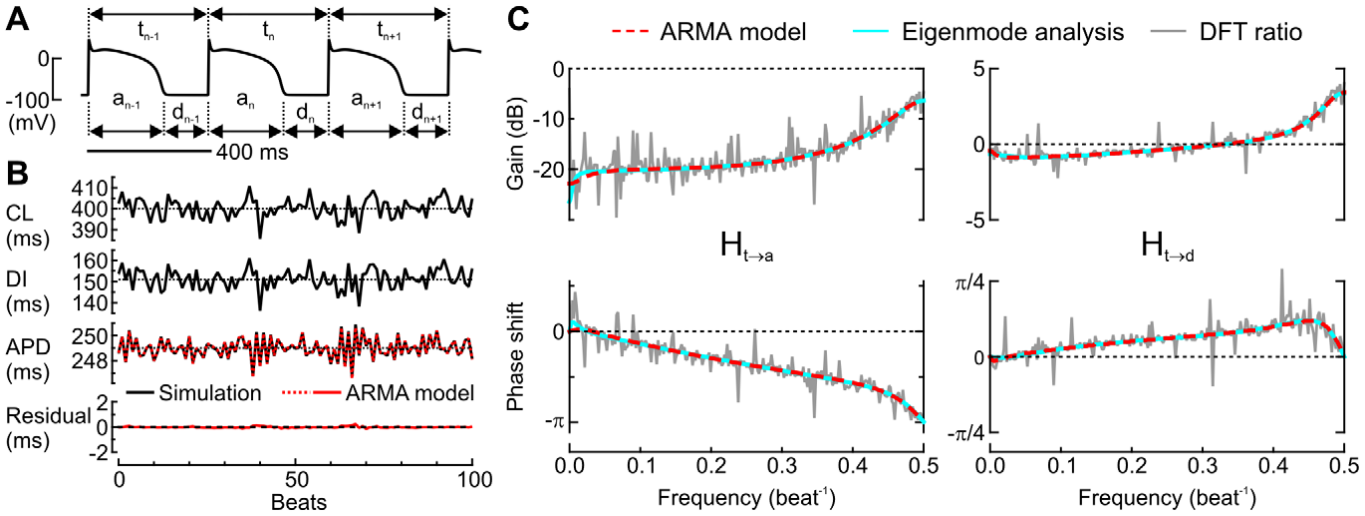
Example analysis in the Ca^2+^-driven model with positive Ca^2+^ to APD coupling. The model was paced at cycle lengths (CLs) varying randomly around 400 ms with a SD of 5 ms. *A*: Example APs, together with the convention regarding the numeration of CLs (t), APDs (a) and DIs (d). *B*: CLs, DIs, APDs during 100 consecutive beats, estimation of APD based on a 3^rd^ order ARMA model (Eq. 18), and residual. The identified coefficients were: α_1_ = −0.2329, α_2_ = −0.7401, α_3_ = 0.0395, and β_1_ = 0.1351, β_2_ = −0.1058, β_3_ = −0.0293. The poles of the ARMA model were *p*_1_ = −0.780, *p*_2_ = 0.053, *p*_3_ = 0.961. *C*: Comparison of the transfer functions H_t→a_ and H_t→d_ (gain and phase shift) derived using eigenmode analysis, computed from the ARMA model coefficients and obtained directly from ratios of discrete Fourier transforms (DFTs, on 512 consecutive cycles), respectively.

In the example illustrated in Figure 3, the Ca^2+^-driven cell model with positive Ca^2+^ to APD coupling was first paced at a constant BCL of 400 ms and exhibited a stable 1∶1 response at steady state. Subsequently, the cell was paced at CLs varying randomly with a SD of 5 ms around 400 ms. Figure 3 *A* depicts simulated APs and Figure 3 *B* represents successive CLs, DIs, and APDs. As shown in Figure 3 *B*, the series of APDs during random pacing was well fitted by a 3^rd^ order ARMA model, accounting for >99% of APD variance with a residual variance <1%. The pole of the ARMA model closest to −1 was −0.780, very near to λ_alt_ = −0.790 computed using eigenmode analysis.

Figure 3 *C* compares the transfer functions H_t→a_ and H_t→d_ of the ARMA model with those derived using eigenmode analysis and those calculated directly from the ratios of the Fourier transforms of the APD, DI and CL time series. The three computations were all in agreement. Furthermore, the transfer functions obtained with the ARMA model matched almost exactly those predicted using eigenmode analysis, except at low frequencies <0.05 beat^−1^.

These transfer functions represent the model behavior in terms of gain and phase shift in the frequency domain. The negative gain of H_t→a_ indicates that at a mean CL of 400 ms, variations of APD are small relative to variations of CL, and thus that the effects of APD restitution are moderate. Conversely, the gain of H_t→d_ around 0 shows that CL variations translate primarily to DI variations. However, the increase of H_t→d_ to +3.5 dB at f = 0.5 beat^−1^ indicates that DI variations at higher frequencies are actually amplified, revealing the propensity of the model to generate alternans.

### Comparative analysis of alternans markers in the three model versions

**Figure 4:**
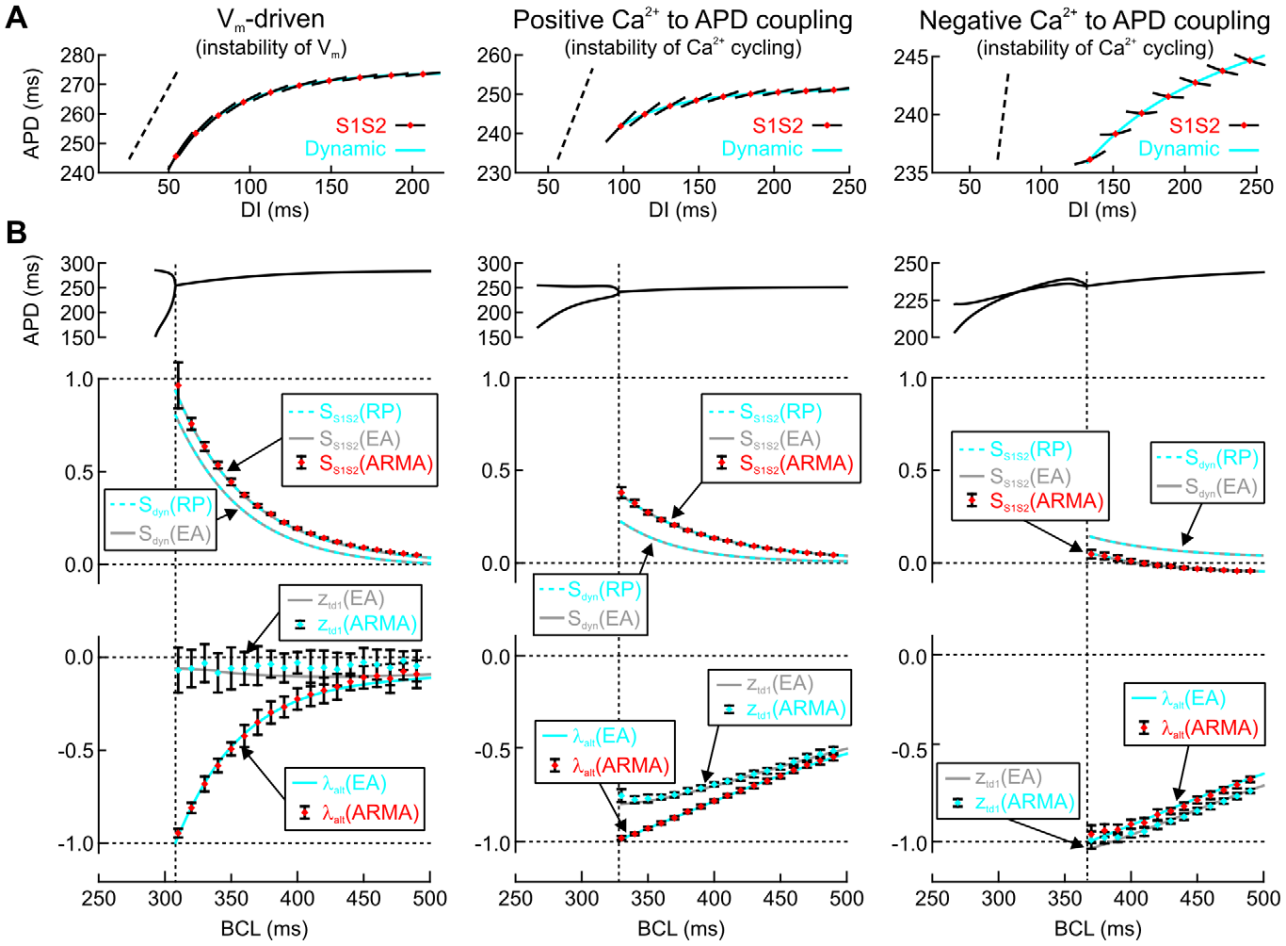
Behavior of alternans markers in the three model versions. *A*: Restitution portraits of APD vs. DI established using a dynamic restitution protocol combined with S1S2 protocols (close-ups of Figure 2). Each portrait consists of a dynamic restitution curve and a family of S1S2 restitution curves at various BCLs. These S1S2 restitution curves are only shown in the neighborhood of their intersections with the dynamic restitution curve (operation points, red diamonds). The oblique dotted lines denote a slope of 1. *B*: APD bifurcation diagrams (close-ups of Figure 1 *A*), conventional slopes (S_S1S2_ and S_dyn_) and transfer function markers (alternans eigenvalue λ_alt_ and first zero of the transfer function H_t→d_ z_td1_) as a function of BCL, identified using eigenmode analysis (EA), conventional restitution protocols (RP), and ARMA model identification during stochastic pacing (SD of CL: 5 ms), respectively. ARMA model identification was conducted on series of 30 successive cycles using a 3^rd^ order model; data points with error bars represent mean±SD for *n* = 37 simulations. Vertical dotted lines denote the BCL at which alternans appears.

Figure 4 shows the restitution portraits of the three model versions in more detail and compares the behavior of the S1S2 and dynamic restitution slopes (S_S1S2_ and S_dyn_, respectively) and the markers λ_alt_ (alternans eigenvalue) and z_td1_ (first zero of the transfer function H_t→d_) as a function of BCL. The restitution portraits (Figure 4 *A*) reflect the clearly distinct restitution dynamic in the three model versions. In these portraits, it is once more apparent that dynamic and S1S2 restitution curves are not equivalent, a behavior which reflects memory [17], [20].

Figure 4 *B* first explores the behavior of the different markers extracted with the different methods, as BCL approaches the bifurcation to alternans. In all versions of the model, the conventional slopes S_S1S2_ and S_dyn_ derived using eigenmode analysis were indistinguishable from those obtained using conventional restitution protocols. However, both S_S1S2_ and S_dyn_ were poor predictors of alternans as they always were <1 at the critical BCL at which alternans appeared. In contrast, λ_alt_ was always exactly −1 at the onset of alternans as predicted by theory. The behavior of z_td1_ further reflects the different dynamic mechanisms governing restitution. While z_td1_ remains near 0 in the V_m_-driven model, it follows a nearly parallel course to λ_alt_ in the Ca^2+^-driven models, but with z_td1_ > λ_alt_ for positive Ca^2+^ to APD coupling and z_td1_ < λ_alt_ for negative Ca^2+^ to APD coupling.

Figure 4 *B* then explores the ability of stochastic pacing combined with ARMA model fitting to estimate these different markers. This approach permitted the robust estimation of both λ_alt_ in all three versions of the ventricular cell model, and this estimation was excellent at regimes when λ_alt_ was close to −1 (a feature which is essential for the practical prediction of alternans). Similarly, z_td1_ could be accurately estimated when it was larger than >0.5 (in absolute value). When these markers were close to 0 (e.g., in the V_m_-driven model), the estimation became less reliable, in agreement with the notion that poles and zeros near 0 exert only a small influence on the dynamics of a time series, which renders their identification difficult [32], [37].

The combination of stochastic pacing and ARMA model fitting also permitted the reliable estimation of S_S1S2_ according to Eq. 20 without actually conducting an S1S2 protocol. However, the estimates of S_dyn_ with the ARMA model according to Eq. 21 were prone to a large variability (not shown). This is explained by the fact that H_t→a_ and H_t→d_ obtained with the ARMA model do not capture the transfer functions with a sufficient reliability at very low frequencies (see Figure 3 *C*). Because S_dyn_ is given by H_t→a_ and H_t→d_ at *f* = 0 (Eq. 14), the estimation of S_dyn_ with ARMA model fitting is thus prone to be less robust.

### Aspect of the transfer functions at pacing regimes closer and closer to the bifurcation to alternans

**Figure 5:**
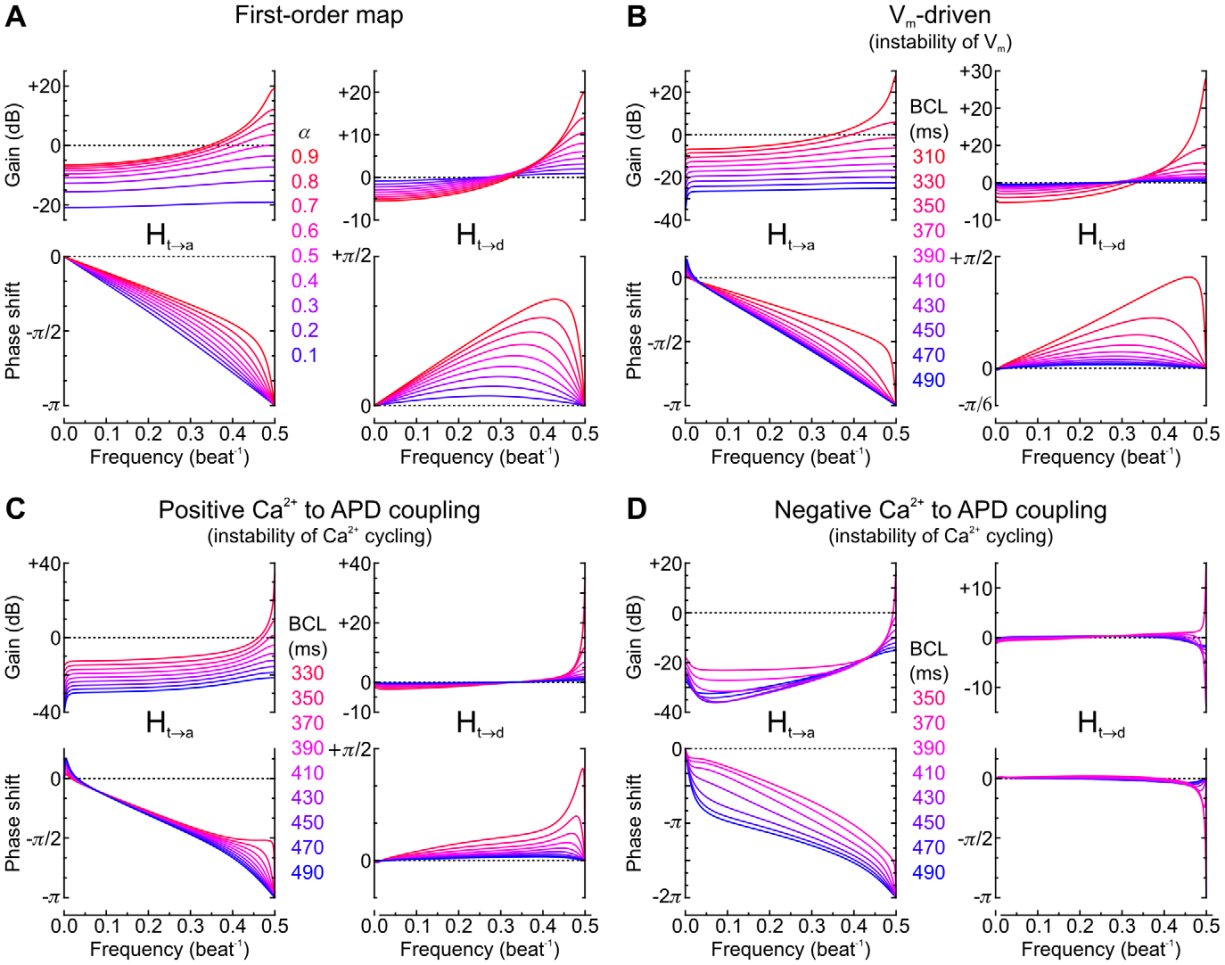
Aspect of the transfer functions at regimes closer and closer to the bifurcation to alternans. *A*: Transfer functions H_t→a_ and H_t→d_ in a theoretical first-order memoryless model given by APD*_n_* = *f*(DI*_n_*_−1_), with α being the slope of *f*. *B*, *C* and *D*: Transfer functions H_t→a_ and H_t→d_ in the three versions of the Sato et al. model, computed by eigenmode analysis at various BCLs.

Representations of frequency response spectra are intuitively easier to interpret than corresponding sets of poles and zeros. Therefore, we investigated how the aspect of the transfer functions H_t→a_ and H_t→d_ behaves at regimes closer and closer to the bifurcation to alternans. As a reference, we first computed these transfer functions for the classical first-order memoryless restitution function APD_n_ = *f*(DI_n−1_) [9] with a slope α = d*f*/dDI at the operation point. These transfer functions are H_t→a_ = α/(*z*+α) and H_t→d_ = z/(*z*+α), with *z* = e^2π*if*^, as can be deduced from Eqs. 12 and 15–19 and as we showed previously [24]. They are represented in Figure 5 *A* for α ranging from 0.1 to 0.9. The transfer functions in the three versions of the Sato et al. model are then shown in Figure 5 *B*–*D* for BCLs approaching the bifurcation to alternans.

From Figure 5, it is apparent that the relationship between stochastic variations of CL and resulting variations of APD and DI is nonlinear, non-additive and frequency-dependent. At CLs far from the alternans regime (darker purple curves), restitution was less involved and CL variations translated primarily into variations of DI, while APD variations were comparatively small. This is reflected by a gain close to 0 dB for H_t→d_ and a negative gain (attenuation) for H_t→a_. However, for all models and at regimes progressively closer and closer to the bifurcation to alternans (lighter redder curves), CL variations resulted in a more and more positive gain for both H_t→d_ and H_t→a_ at frequencies >0.4 beat^−1^, with a peak at 0.5 beat^−1^. This observation can be interpreted as an increasing propensity to alternans. This gain reached values up to 20 dB, which corresponds to amplification by a factor of 10. Thus, in regimes close to the development of alternans, variations of APD and DI may reach a level which is comparatively one order of magnitude higher compared to variations of CL.

The behavior of the transfer functions in the V_m_-driven model (Figure 5 *B*) was qualitatively similar to that in the first-order model, suggesting a low level of memory in the V_m_-driven Sato et al. model. In contrast, the behavior in the Ca^2+^-driven models was clearly different. With positive Ca^2+^ to APD coupling (Figure 5 *C*), the curves appear skewed towards the right. With negative Ca^2+^ to APD coupling (Figure 5 *D*), the aspect is fundamentally different. First, at *f* = 0.5 beat^−1^, the phase shift of H_t→a_ is −2π instead of −π, a difference explained by the presence of a zero (*z*_td1_) more negative than λ_alt_. Second, a singularity appears at *f* = 0.5 beat^−1^ in H_t→d_ (abrupt change in polarity) when this zero leaves the unit circle at −1 (see Figure 4 *B*). Thus, all these transfer function “portraits” are able to picture and reveal the dynamical differences regarding both the propensity to alternans generation and memory in the different models.

Similar to Figure 3, all these transfer functions could be estimated using the stochastic pacing protocol and ARMA model identification, with small deviations in the low frequency range <0.05 beat^−1^. This range corresponds to time scales of >20 beats and is thus determined by long-lasting effects of memory (poles and zeros near +1), which cannot be captured accurately by the ARMA model. These effects manifest themselves in Figure 5 *B*–*D* as inflections of both the gain and the phase shift curves at *f*<0.05 beat^−1^.

### Prediction of the onset of alternans during a ramp decrease of BCL

**Figure 6:**
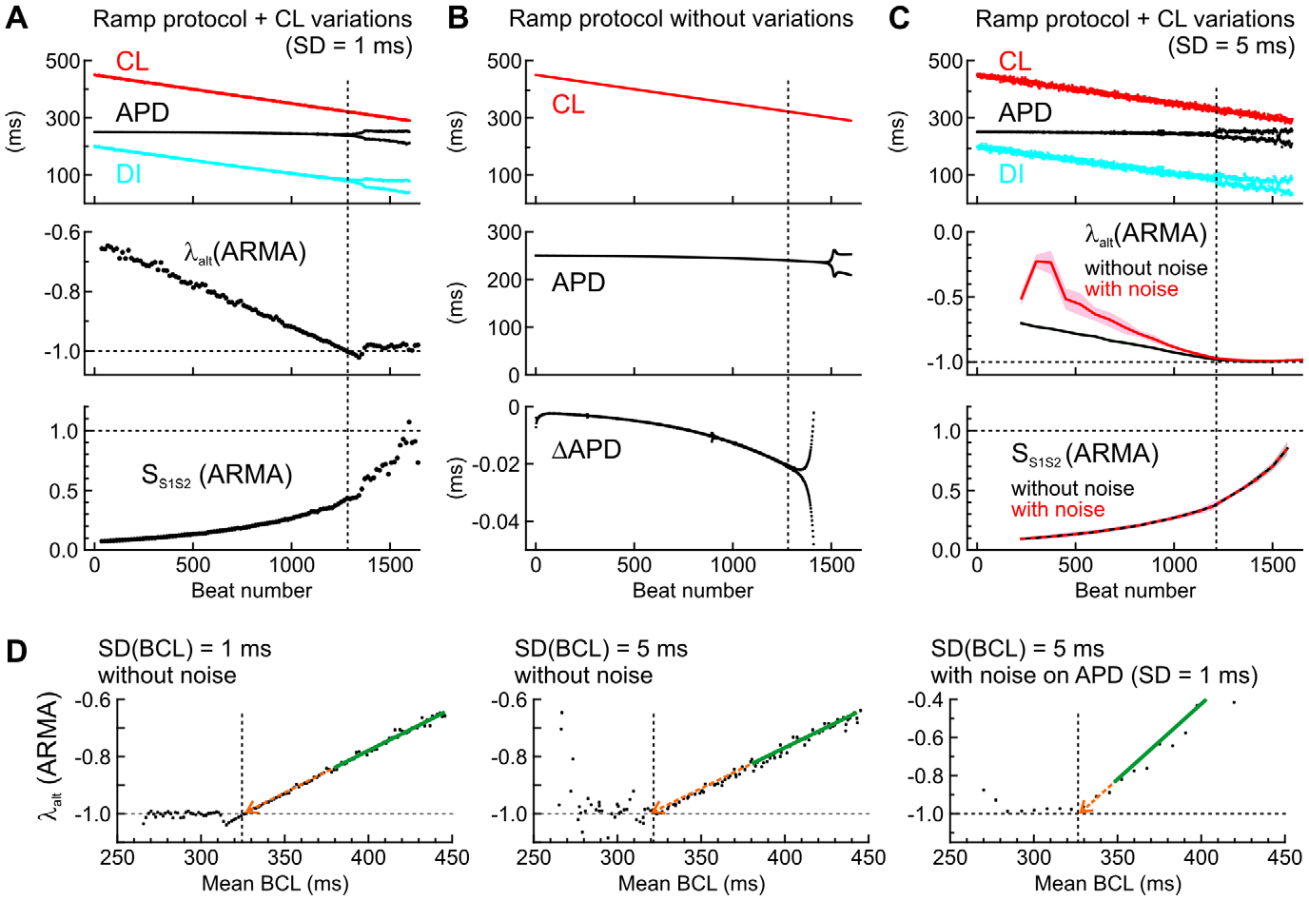
Predicting alternans in the Ca^2+^-driven model with positive Ca^2+^ to APD coupling during ramp pacing. *A*: CL, APD, DI, λ_alt_ and S_S1S2_ during the ramp decrease of CL with superimposed variations (SD = 1 ms). λ_alt_ and S_S1S2_ were estimated every 15 beats using a 2^nd^ order ARMA model from APDs and CLs in a window spanning the 30 preceding cycles. *B*: CL, APD and the difference series of APD (ΔAPD = APD*_i_* – APD*_i_*_−1_) during the ramp decrease of CL without stochastic variations. *C*: *Top*: CL, APD and DI during the ramp decrease of BCL with superimposed CL variations (SD = 5 ms). *Middle* and *bottom*: Analysis of λ_alt_ and S_S1S2_ in *n* = 10 simulations, with and without adding a Gaussian error (SD = 1 ms) to APD, simulating experimental measurement error. λ_alt_ and S_S1S2_ were estimated every 75 beats using a 2^nd^ order ARMA model from APDs and CLs in a window spanning the 150 preceding cycles. Data are presented as mean (bold lines) ± SD (shaded areas). In all panels, vertical dotted lines indicate the onset of macroscopic alternans (in *A* and *C*) and of micro-alternans (in *B*). *D*: Prediction of alternans by analyzing λ_alt_ vs. mean BCL in the windows in which λ_alt_ was evaluated. *Left*: using a SD of BCL of 1 ms (windows of 30 cycles). *Middle*: using a SD of BCL of 5 ms (windows of 30 cycles). *Right*: using a SD of BCL of 5 ms, after adding a Gaussian error on APD (SD = 1 ms; windows of 150 cycles). The green lines represent linear regression on data points with λ_alt_>−0.85, and the dotted orange arrows represent the extrapolation of these regression lines towards λ_alt_ = −1. The occurrence of alternans is marked with vertical dotted lines.

The analyses presented above were conducted in stationary regimes, for which mean BCL and cellular properties did not evolve with time. However, in electrophysiological experiments, the propensity to alternans is typically assessed by decreasing BCL (either stepwise or progressively) until alternans appears. Therefore, we examined whether determination of λ_alt_ using ARMA model identification would permit to anticipate the onset of alternans during a slow decrease of BCL. Results with the Ca^2+^-driven model with positive Ca^2+^ to APD coupling are shown in Figure 6.

In Figure 6 *A*, the model was paced using a protocol consisting of CLs decreasing progressively (−0.1 ms/beat) to which random Gaussian variations (SD: 1 ms) were added. The resulting series of APDs and CLs were then segmented in windows of 30 cycles with an overlap of 15 cycles, and λ_alt_ as well as S_S1S2_ were estimated from the data in each window using a 2^nd^ order ARMA model. In the illustrated example, λ_alt_ progressively approached −1 and the onset of alternans coincided with the moment when λ_alt_ reached −1 (vertical dotted line). Thus, observing the course of λ_alt_ as it gets closer to −1 allows anticipating alternans.

Figure 6 *A* shows once more that S_S1S2_ is a poor predictive marker, as its value was only 0.4 at the onset of alternans. In Figure 6 *B*, the model was paced using the same ramp protocol as in Figure 6 *A*, but without adding random CL variations, i.e., using a control ramp protocol without stochastic variations. Although manifest APD alternans appeared later than in Figure 6 *A*, the difference series of APD (ΔAPD = APD*_i_* – APD*_i_*_−1_) reveals that microscopic alternans (micro-alternans) actually appeared at the same moment as anticipated in Figure 6 *A* from the behavior of λ_alt_. Similar results were obtained with the V_m_-driven model and the Ca^2+^-driven model with negative Ca^2+^ to APD coupling.

In an experimental setting, APD is always subject to measurement error. To evaluate how our analyses would be influenced by measurement error, we conducted 10 simulations as in Figure 6 *A* and added a random Gaussian error on the APD time series before the evaluation of λ_alt_ and S_S1S2_. In these simulations, the SD of the random CL deviations was increased to 5 ms and the SD of the error added to APD was 1 ms. As illustrated in Figure 6 *C*, adding noise to the APD time series resulted in an underestimation of λ_alt_ (in absolute value) and a larger variability of the estimates, which necessitated increasing the number of cycles used for ARMA model fitting to 150. However, the estimation of λ_alt_ became progressively more accurate as it approached −1, and extrapolating the time course of λ_alt_ towards its intercept with the line λ_alt_ = −1 permitted to anticipate the onset of alternans as in the control situation without adding noise to APD. Interestingly, the estimation of S1S2 was not influenced by measurement noise.

It is also informative to analyze λ_alt_ as a function of BCL, as done in Figure 6 *D* for the data presented in Figure 6 *A* and *C*. In this analysis, linear regression of λ_alt_ vs. BCL was conducted for data points with λ_alt_>−0.85. By extrapolating the regression line to λ_alt_ = −1, it was possible to anticipate the BCL at which alternans developed.

### Prediction of alternans during progressive I_Ks_ block

**Figure 7:**
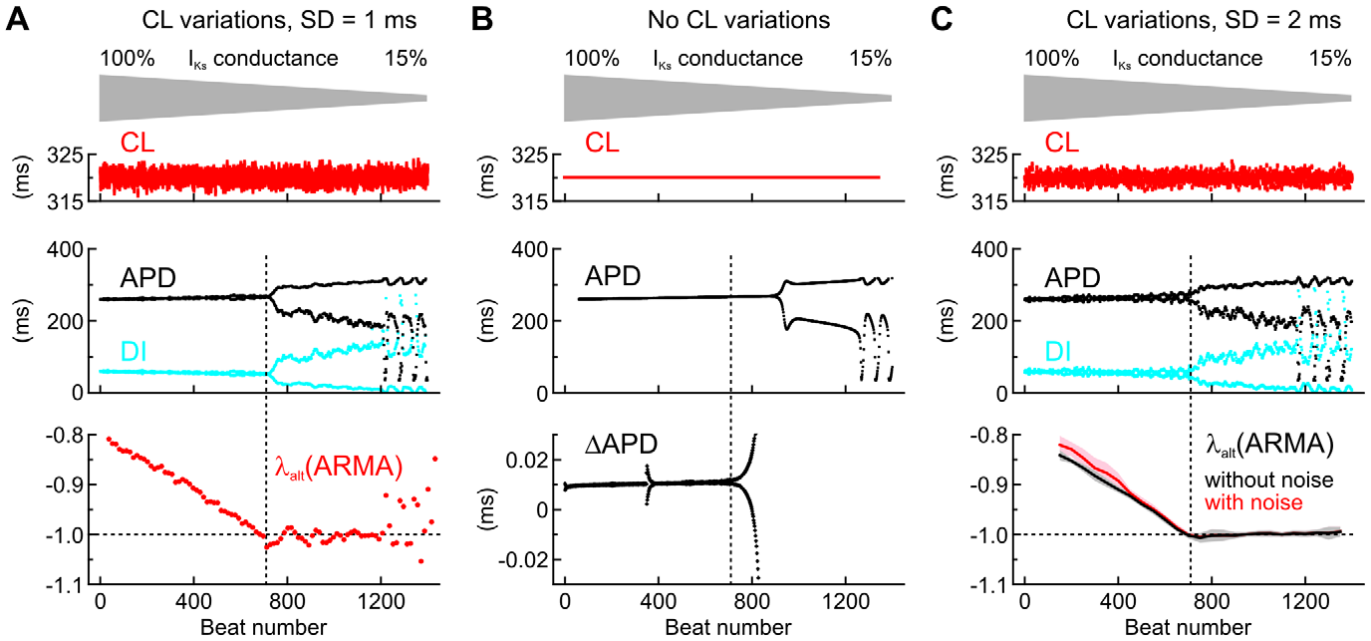
Predicting alternans in the V_m_-driven model during a progressive decrease of I_Ks_ conductance. *A*: CL, APD, DI and λ_alt_ during stochastic pacing (mean CL: 320 ms; SD of variations: 1 ms). λ_alt_ was estimated every 15 beats using a 2^nd^ order ARMA model from APDs and CLs in a window spanning the 30 preceding cycles. *B*: CL, APD and the difference series of APD (ΔAPD) when pacing at a CL = 320 ms without variations. *C*: *Top*: CL, APD and DI during stochastic pacing (mean CL: 320 ms; SD of variations: 2 ms). *Middle* and *bottom*: Analysis of λ_alt_ in *n* = 10 simulations, with and without adding a Gaussian error (SD = 1 ms) to APD, simulating experimental measurement error. λ_alt_ was estimated every 75 beats using a 2^nd^ order ARMA model from APDs and CLs in a window spanning the 150 preceding cycles. Data are presented as mean (bold lines) ± SD (shaded areas). In all panels, vertical dotted lines indicate the onset of macroscopic alternans (in *A* and *C*) and of micro-alternans (in *B*).

We then evaluated whether estimating λ_alt_ during a slow change of cellular properties would also allow anticipating the onset of alternans. The V_m_-driven model was paced at a stationary rate (BCL = 320 ms) and the conductance of the slow delayed rectifier K^+^ current (I_Ks_) was progressively reduced at a rate of 0.2% per second, starting from its nominal value of 100%, to mimic the slow application of an I_Ks_ channel blocker. Figure 7 *A* depicts the behavior of APD, DI and λ_alt_ (estimated in windows of 30 cycles as in Figure 6 *A*) during stochastic pacing (mean CL: 320 ms; SD of CL: 1 ms). As in Figure 6 *A*, λ_alt_ progressively approached −1 and alternans appeared when λ_alt_ reached −1. This indicates that observing the course of λ_alt_ as it gets closer to −1 may also allow anticipating alternans during pharmacologic interventions. Figure 7 *B* represents the control situation, in which the model was paced at a constant CL of 320 ms without random variations, but with the same decrease of I_Ks_ conductance. Manifest alternans appeared later than in Figure 7 *A*, but micro-alternans (visible in the ΔAPD series) appeared at the same moment as anticipated from the evolution of λ_alt_.

The sensitivity of the estimation of λ_alt_ on noise added to the APD time series was investigated in Figure 7 *C* with an approach similar to that used in Figure 6 *C*. A random Gaussian error was added on the APD time series before the evaluation of λ_alt_ and the simulation was repeated 10 times. In these simulations, the SD of the random CL deviations was 2 ms and the SD of the error added to APD was 1 ms. The number of cycles used for ARMA model fitting was adjusted to 150. Adding noise to the APD time series resulted in a slight underestimation of λ_alt_ (in absolute value) but did not affect the time of its intercept with the line λ_alt_ = −1. Thus, predicting the onset of alternans was not precluded by the noise added to APD.

### Superiority of ARMA model identification compared to a time domain analysis

**Figure 8:**
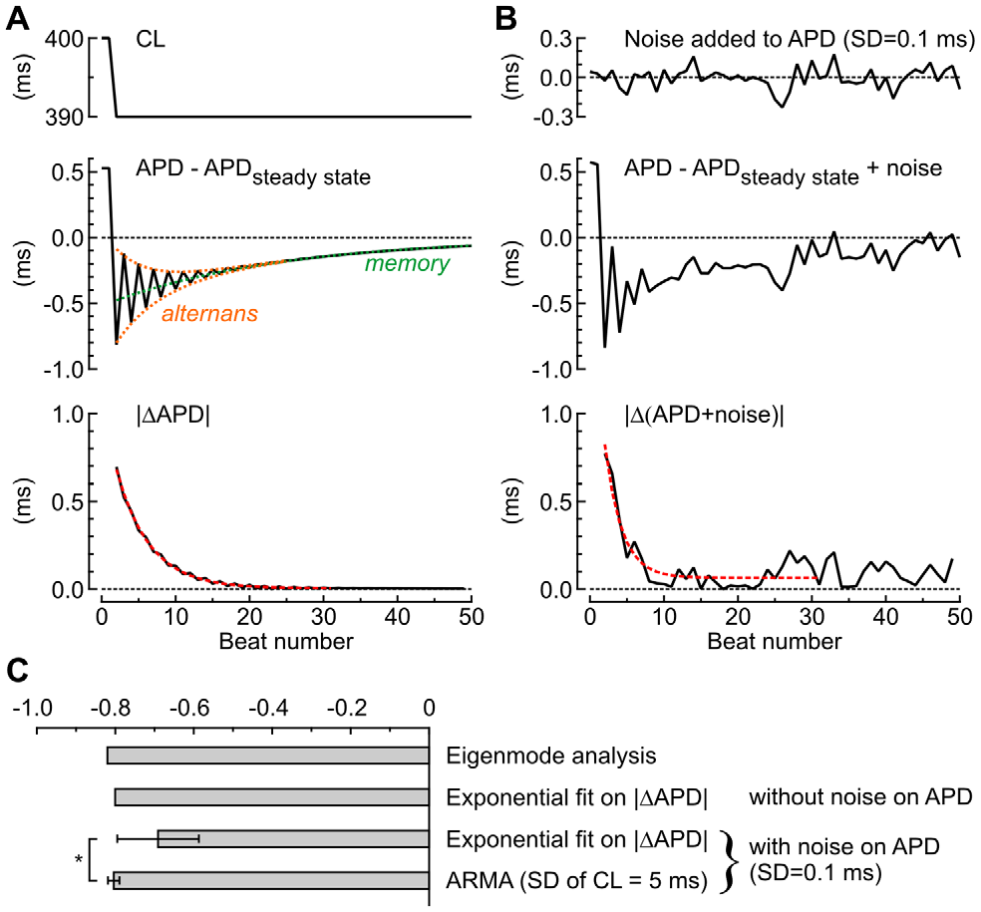
Superiority of ARMA model identification during stochastic pacing vs. a time domain analysis after a step change in BCL. *A*: Response of the Ca^2+^-driven model with positive Ca^2+^ to APD coupling to a step decrease of BCL from 400 to 390 ms. *Top*: Pacing protocol. *Middle*: Difference between APD and steady state APD at BCL = 390 ms, showing transient decaying alternans followed by an exponential convergence to steady state. Orange and green dotted lines denote the components determined by the alternans and memory eigenmodes, respectively. *Bottom*: Absolute value of the APD difference series, reflecting the decay of the alternans eigenmode. The red curve illustrates the best fit with an exponential function, yielding λ_alt_ = −0.800. *B*: Same data and analysis as in *A*, but with a Gaussian noise added on APD (SD = 0.1 ms, *top panel*), yielding λ_alt_ = 0.643. *C*: Comparison of λ_alt_ obtained with different methods, with eigenmode analysis serving as control. In the presence of noise added to APD, stochastic pacing and ARMA model identification was superior to the time domain analysis with exponential fitting (n = 37; * p<0.05, two-tailed Fisher-Snedecor's *F*-test on variance).

To investigate whether ARMA model identification during stochastic pacing offers a significant advantage over a simpler time domain analysis consisting of quantifying the decay of APD oscillations following a perturbation, we examined the response of the Sato et al. model to a step change of BCL. An example is illustrated in Figure 8 *A* for the Ca^2+^-driven model with positive Ca^2+^ to APD coupling after a step decrease of BCL from 400 to 390 ms. The step decrease of BCL caused transient decaying APD alternans, followed by an exponential convergence of APD to its new steady state at BCL = 390 ms. These two patterns reflect the alternans and memory eigenmodes, respectively. To quantify the decay of the alternans eigenmode, an exponential function was fitted to the absolute value of the APD difference series (|ΔAPD|). The time constant of this function provided an estimate of λ_alt_ of −0.800, which was close to the control value of −0.820 derived using eigenmode analysis. However, as shown in Figure 8 *B*, this estimation was compromised when noise was added to APD (SD = 0.1 ms). In Figure 8 *C*, this time domain method is compared statistically to ARMA model identification during stochastic pacing (SD of APD: 5 ms; 3^rd^ order ARMA model, identification over 30 cycles). In the presence of noise, the variability of λ_alt_ estimates was significantly smaller for ARMA model identification during stochastic pacing. Similar results were obtained at other BCLs and for the two other versions of the cell model. Thus, in the presence of noise, ARMA model identification is more robust than quantification of the exponential decay of APD alternation following a step decrease of BCL.

## Discussion

Alternans is a clinically relevant phenomenon leading to dispersion of refractoriness, which precipitates conduction block and reentrant arrhythmias [2]–[4]. The prediction of cardiac arrhythmias and the understanding of their underlying mechanisms have always represented a great challenge in both research and cardiology practice. For these reasons, understanding electrical restitution and alternans has been the topic of numerous studies for more than four decades, starting with the classical one-dimensional iterated map model of Nolasco and Dahlen [9]. The dynamic mechanisms leading to alternans are manifold: they involve not only complex interactions between ion currents and Ca^2+^ handling at the cellular level, but also intercellular interactions at scales ranging from tissue to the whole organ [2]. Because of this multiplicity of mechanisms and the complexity of cardiac dynamics, predicting alternans and arrhythmias still remains a difficult endeavor.

### A new unified framework for restitution, alternans and memory

In the present study, we revisit restitution by examining it in a generalized framework based on eigenmode analysis, a sound mathematical approach for the characterization of dynamical systems. Previous studies based on eigenmode analysis [28], [29] have shown that in cardiac electrophysiology, alternans and memory are actually the two faces of the same dynamics. By looking into eigenmode analysis in the frequency domain, we first showed that in the linear limit, the eigenmode description of a cardiac cell is equivalent to the description using a memory model of cardiac restitution. Both descriptions can be understood in terms of transfer functions. In the frequency domain, memory and alternans can then be regarded as the two extremes on the frequency axis, which ranges from 0 to 0.5 beat^−1^. Based on engineering notions of signal processing, we then devised a practical method to determine these transfer functions together with their poles and zeros using only time series of CLs, APDs and DIs. Our key finding is that the propensity to alternans can be quantified and monitored and thus the onset of alternans can be anticipated using the eigenvalue (first pole) λ_alt_ obtained via ARMA model identification during pacing at intervals varying randomly. The results of the computer simulations, conducted with models exhibiting three fundamentally different ionic mechanisms of alternans, not only support the general validity of this approach but also suggest that it may also be applied in non stationary regimes such as during a slow acceleration of the (average) pacing rate or the progressive application of a drug. Our approach is general because it can be applied to any model based on a biophysical description of ion currents as well as any higher-dimensional iterated map model of cardiac dynamics, such as the models of Vinet et al. [10], Chialvo et al. [13] or Qu et al. [38], for which eigenmode analysis can also be conducted and thus the transfer functions determined.

Our approach develops its full strength when pacing at randomly varying intervals is considered. Our simulations provide the proof of principle that stochastic pacing permits the estimation of the transfer functions H_t→a_ and H_t→d_ and thus of their first pole as a marker for the propensity to alternans. It is worth to note that in our simulations, ARMA model identification was excellent in estimating S_S1S2_, even in the presence of measurement noise. Thus, the S1S2 restitution slope can actually be determined by stochastic pacing without the need to conduct an S1S2 protocol. However, the performance of ARMA model identification to estimate S_dyn_ was low. This observation is explained by the fact that the determination of S_dyn_ (using Eq. 21) is exquisitely sensitive to errors in the determination of the higher order coefficients of the ARMA model and that these errors cannot be decreased by increasing the order of the ARMA model. Indeed, in our simulations, >99% of APD variance was already described with a model of order 2 or 3, and increasing this order provided neither a better description of the dynamics, neither a higher reliability in computing λ_alt_ or restitution slopes. This indicates that the dynamics of the Sato et al. model, which has 16 variables, can be well represented by a lower dimensional model of order 2 or 3 in near stationary regimes. This also explains that the estimation of S_dyn_ using ARMA model identification is prone to a large variability, and thus that it would not be superior to a conventional pacing protocol in a practical experimental setting.

In this study, we used APD and DI as the system's output time series. It must be noted that our approach can also be used to derive the transfer function between pacing cycle lengths and any other output parameter such as the peak Ca^2+^ transient or the peak of a given ion current. Therefore, in an experimental setting, ARMA model identification during stochastic pacing could also be applied on series of peak Ca^2+^ transients, or, a fortiori, on local conduction velocities or mechanical parameters (e.g., peak force or shortening). Our approach also offers the advantage to be versatile. For example, exploring the frequency response of the Ca^2+^ transient in addition to the response of APD may uncover additional insights regarding the primary cause of alternans (voltage vs. Ca^2+^ driven), which may be pertinent in determining appropriate clinical therapeutic strategies (e.g. conventional pharmacotherapy targeting ion channels vs. new agents that may target the cellular Ca^2+^ handling machinery). This analysis was however beyond the scope of this work.

### Relation and consistency with previous studies

As demonstrated in the Methods section, the classical one-dimensional memoryless map [9] represents a particular case for which the criteria S_dyn_ = 1, S_S1S2_ = 1 and λ_alt_ = −1 are equivalent. However, the equivalence of these criteria breaks down as soon as cardiac dynamics exhibit memory. Memory can thus be defined as any deviation from the first order map behavior. Thus, the notion of memory clearly explains why, in a more general setting, any prediction of alternans based on S_dyn_ or on S_S1S2_ [17], [18] should not be expected to be reliable.

In a theoretical study, Tolkacheva et al. [25] derived criteria for alternans and stability based on measuring dynamic and S1S2 restitution slopes in an iterated map model given by APD*_n_*_+1_ = *f*(APD*_n_*,DI*_n_*). The authors then generalized their analysis to mapping models with an arbitrary amount of memory [21] (corresponding to Eq. 15). The mapping models were investigated in the time domain using the perturbed downsweep protocol. We note that our framework is fully consistent with their time domain analyses, as it yields, for example, an equivalent result for S_dyn_. However, our approach provides additional insights in the Z and frequency domains and links restitution to eigenmode analysis. As a principle, frequency domain analysis permits to understand cardiac dynamics in response to any arbitrary sequence of pacing intervals, including stochastic pacing and pacing at cycle lengths varying in an oscillatory manner.

In this latter context, the recent studies of Wu and Patwardhan deserve attention. To demonstrate memory effects, these investigators paced a ventricular cell [26] or a mathematical cell model [39] while controlling the DI and varying it as a sinusoidal function with a period of 100 beats. This sinusoidal variation resulted in hysteresis of APD vs. DI, i.e., in a phase shift between both. Because a sinusoidal pacing protocol can be regarded as probing the transfer functions at the corresponding frequency (*f* = 0.01 beat^−1^), memory effects should become apparent at this frequency in graphical representations of transfer functions. Accordingly, at frequencies ≤0.01 beat^−1^, the three versions of the Sato et al. model are characterized by manifest phase shifts (Figure 5 *B*–*D*), whereas the phase shift is vanishingly small in the memoryless first-order model (Figure 5 *A*). While a sinusoidal pacing protocol thus represents a suitable approach to probe memory, the advantage of the stochastic pacing protocol is that it examines all frequencies at the same time, thus probing both alternans and memory.

### Perspectives, limitations and challenges

Stochastic pacing and ARMA model identification would be straightforward to implement in any electrophysiological apparatus. Therefore, our approach could readily be translated to *in vitro* and *in vivo* models, opening the perspective of new diagnostic approaches during clinical investigation of heart rhythm disorders. In the future, one could for example envision stochastic pacing for clinical electrophysiological testing as a built-in extension to cardiac mapping systems or implanted defibrillators for the purpose of risk-stratification.

Obviously, our theoretical framework must withstand the challenge of experimental validation. Because experimental data such as APD measurements are always subject to measurement error and because APD variability may also result from the stochastic gating of ion channels [40], it will first be necessary to carefully optimize the SD of stochastic pacing variations, the number of cycles used for ARMA model identification and the order of the ARMA model. Our simulations suggest that our approach performs well as long as the system remains near its linear limit. If the SD of CL is set to be too large, our approach will eventually be limited by nonlinearities in the system and some stimuli may fall in the refractory period. To minimize the intrinsic variability of APD, it may then be appropriate to use small pieces of cardiac tissue in which this intrinsic variability is strongly decreased by gap junctional coupling between individual cells [40]. It will also be necessary to evaluate the effects of other sources of variability in both experiments and further computer simulations. Nevertheless, our computational results indicate that our approach should be robust in predicting the onset of alternans.

From a theoretical point of view, it will also be necessary to extend the theory to multicellular systems in order to understand the influences of intercellular interactions, the effects of conduction velocity restitution [41], [42] and the consequences of multidimensional phenomena such as wavefront curvature [43]. In particular, it has been shown in spatially extended systems that electrotonic interactions can exert a large influence on the occurrence of alternans and the APD restitution slope at which alternans occurs [16], [23]. Furthermore, the occurrence of alternans can be significantly modulated by steep conduction velocity restitution slopes [15]. These aspects will require a careful computational evaluation. Such computational studies will permit to understand the possibilities and limitations of our framework in greater detail, and they are expected to provide additional insights into dynamical phenomena emerging at multicellular scales, such as spatially discordant alternans [41], [42].

### Conclusion

In conclusion, stochastic pacing combined with ARMA model identification represents a novel frequency domain approach to study cardiac dynamics. This approach should be applicable experimentally for the accurate evaluation of the propensity to alternans and the prediction of its onset. Because its mathematical foundation does not make any a priori assumptions about the ionic mechanisms of alternans, it pertains to any type of myocardial cell or tissue, irrespective of species, disease status or pharmacological interventions.
